# Metabolic - Bariatric Surgery in Elderly Patients – Safety and Effectiveness in a Single-Center Cohort

**DOI:** 10.1007/s11695-026-08738-7

**Published:** 2026-05-22

**Authors:** Lea Pedarnig, Julia Jedamzik, Larissa Nixdorf, Paula Richwien, Magdalena Mairinger, Christoph Bichler, Felix Benedikt Langer, Lisa Gensthaler, Michael Krebs, Gerhard Prager, Daniel Moritz Felsenreich

**Affiliations:** 1 Department of General Surgery, Medical University of Vienna, Vienna, Austria; 2 Department of Internal Medicine III, Medical University of Vienna, Vienna, Austria

**Keywords:** Metabolic bariatric surgery, RYGB, OAGB, Sleeve, SADI-S, Elderly patients, Age 65 years

## Abstract

**Introduction:**

Overweight and obesity have been steadily increasing worldwide. Metabolic/bariatric surgery (MBS) is the most effective treatment for obesity and its associated medical conditions. Obesity complications increase with age, however, older patients face a higher perioperative risk necessitating a clear risk-benefit balance. This study evaluated weight-loss outcomes, remission of associated medical conditions, and complications in patients aged ≥ 65 years undergoing MBS.

**Methods:**

This retrospective analysis included patients aged ≥ 65 years who underwent MBS at the Medical University of Vienna between 2008 and 2025. Weight and associated medical complications were assessed at baseline and at 1, 2, and 5 years postoperatively. Early (< 30 days) and late complications up to 5 years were recorded.

**Results:**

In total, 111 patients were included. Baseline weight was 125.8 ± 22.3 kg and BMI 45.2 ± 7.4 kg/$\:{\mathrm{m}}^{2}$. At 5 years, mean BMI decreased to 27.5 ± 6.2 kg/$\:{\mathrm{m}}^{2}$ corresponding to a total weight loss of 37.3 ± 14.7%. The prevalence of type II diabetes decreased from 54.1% to 22.9% and arterial hypertension from 87.4% to 56.2%. Early and late complications each occurred in 7.2%.

**Conclusion:**

MBS achieves substantial long-term weight reduction and remission of associated medical conditions. Complication rates are relatively high, underscoring careful patient selection is essential. Larger prospective studies focusing on elderly populations are warranted. Keypoints: MBS in elderly patients is effective in terms of weight loss and remission of associated medical complications. - MBS in elderly patients was associated with a relatively high complication rate. - Deaths within the study period were not associated with the MBS procedure.

## Keypoints

MBS in elderly patients is effective in terms of weight loss and remission of associated medical complications.

MBS in elderly patients was associated with a relatively high complication rate.

Deaths within the study period were not associated with the MBS procedure.

## Introduction

Obesity, defined as a body mass index (BMI) ≥ 30 kg/$\:{\mathrm{m}}^{2},\:$is a chronic multifactorial disease driven by genetic, epigenetic, and environmental factors. Its prevalence continues to rise globally and poses a major financial burden on healthcare systems [1]. Metabolic-bariatric surgery (MBS) provides the most effective long-term therapy for obesity achieving superior weight loss and resolution of obesity-associated medical conditions compared with conservative medical therapy [1, 2]. Sleeve gastrectomy (SG) [2], Roux-en-Y Gastric Bypass (RYGB) [3], One-Anastomosis Gastric Bypass (OAGB), and single-anastomosis duodenoileal bypass with sleeve (SADI-S) are the most frequently performed MBS procedures [3].

Common obesity complications include type 2 diabetes mellitus (DMII), arterial hypertension (HTN), hyperlipidemia (HLP), obstructive sleep apnea syndrome (OSAS), and gastroesophageal reflux disease (GERD) [4]. These obesity complications increase with age and contribute to higher morbidity and mortality [1, 5]. With rising life expectancy, increasing numbers of older patients are seeking MBS [6]. However, elderly patients are at a higher perioperative risk due to frailty, reduced physiological reserves, and increased comorbidity burden [7, 8]. Therefore, a careful risk-benefit evaluation is necessary.

This study aimed to determine whether MBS in patients aged ≥ 65 years is safe and effective regarding weight loss and remission of associated medical complications.

## Methods

### Study Design

This retrospective cohort study included all patients aged ≥ 65 years at the time of the surgery undergoing primary MBS (SG, OAGB, RYGB, SADI-S) at the Medical University of Vienna (MUV) between August 2008 and February 2025. Patients that had previous bariatric procedures or revisional procedures were excluded. None of the patients were operated in an emergency setting or for oncological indications. Nevertheless, based on the individual status of preexisting morbidities, patients had additional preoperative internist and anesthesiologic examinations (e.g. echocardiography, pulmonal functional testing, etc.) in order to estimate the individual risk. The estimated individual risk/benefit was then discussed with the patient, hence, not all elderly patients met the anaesthsiological criteria for a safe MBS procedure. Patients with missing follow-up data were contacted.

Baseline variables included age, sex, anthropomorphic measurements, ideal weight (based on BMI of 25.0 kg/$\:{\mathrm{m}}^{2}$), and excess weight. Follow-up assessments at 1, 2, and 5 years included weight, BMI, total weight loss (TWL), and excess weight loss (EWL). TWL was defined as reduction in body weight relative to the initial body weight in percent. EWL was specified as weight lost relative to the patient’s excess weight, based on the difference between initial weight and ideal body weight at BMI 25 kg/m^2^, in percent.

Obesity-related complications (DMII, HTN, HLP, OSAS) were assessed at all timepoints. Remission was defined as discontinuity of all medications for the individual obesity-related complication. GERD remission was specified as an absence of typical symptoms without PPI intake. Clavien Dindo complications 3b and above requiring endoscopic and surgical interventions in general anesthesia were reported in this study and categorized as early complications (occurring within the first 30 days after surgery) or mid-term complications (from one month up to five years). Further, mortality was recorded.

### Surgical Methods

Detailed descriptions of surgical techniques of the four surgical procedures, SG [9], RYGB [10], OAGB [11] and SADI-S [12] as performed at the MUV have been published previously.

### Statistical Analysis

Data were gathered using Microsoft Excel Version 16.89.1. Statistical analysis was conducted using IBM SPSS Version 31.0.0.0. Due to the low number of patients, descriptive statistics were applied. Therefore, no comparison regarding effectiveness of the different procedures in comparison to one another can be provided. Numeric data are presented using mean and standard deviation and/or median and range or interquartile range (IQR). Categorical data is presented using absolute numbers and relative numbers (%). For changes in body weight and BMI, graphs stratified by the different MBS were used.

## Results

Among 3,370 patients undergoing MBS at the MUV between August 2008 and February 2025, 111 (3.3%) were aged > 65.0 years at the time of the surgery and included in this analysis.

**Fig. 1:**
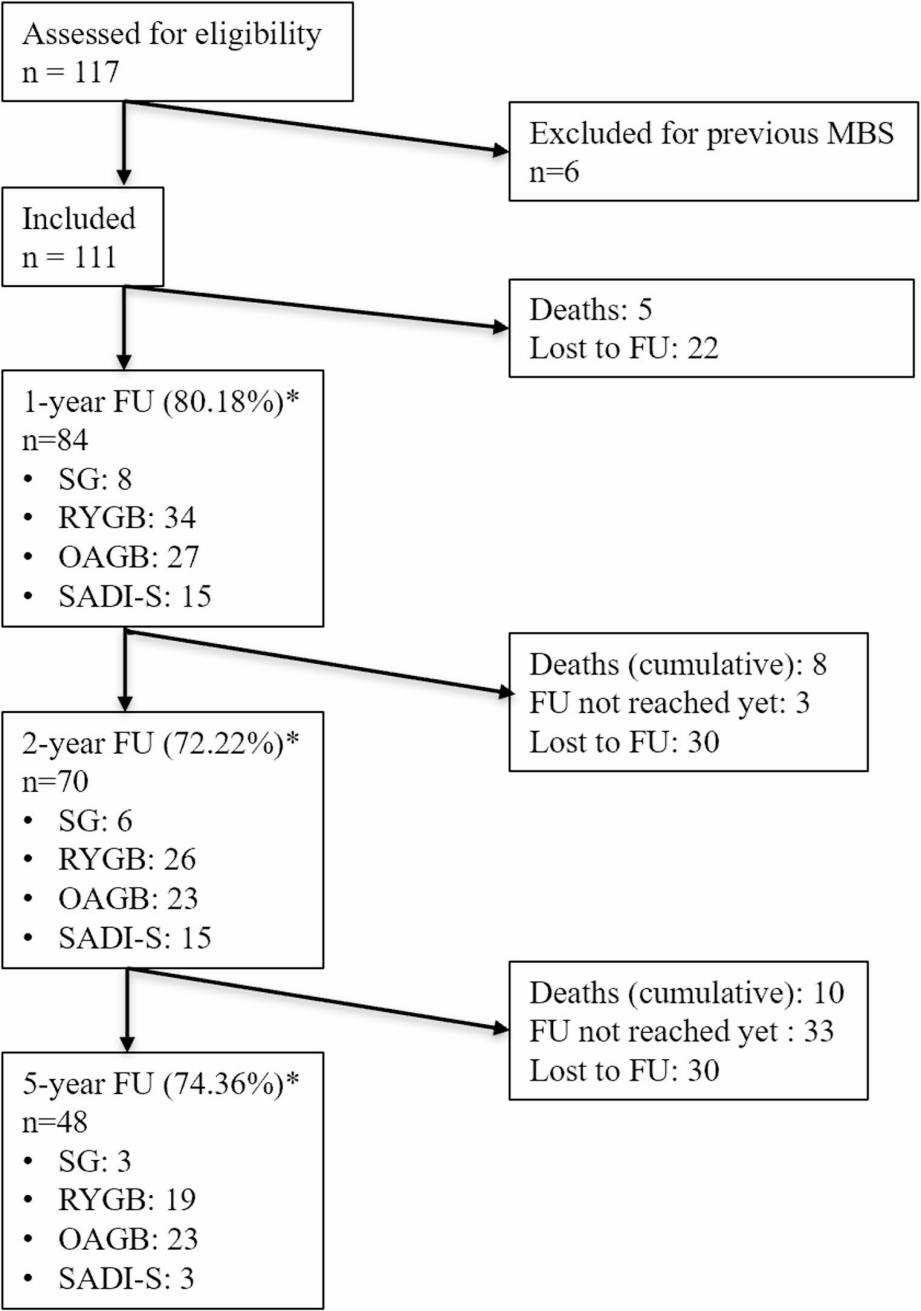
Flow chart of included patients at each timepoint for each procedure. * Follow-up in % (including number of patients at timepoint and dead patients). Abbreviations: Metabolic-bariatric surgery (MBS), Follow Up (FU), Sleeve Gastrectomy (SG); Roux-en-Y Bypass (RYGB), Omega Loop Gastric Bypass (OAGB), Single Anastomosis Duodenoileal Bypass with Sleeve (SADI-S)

Minimal follow-up to be included in this study was one year. Mean follow-up was 3.2 years. Patient follow-up was completed by 89/111 patients (80.2%) after one year, 78/108 (72.7%) after two years, and 58/75 (77.3%) after five years. As some patients missed individual follow-up appointments, each follow-up timepoint must be interpreted individually compared to baseline. Reasons for the missing follow-up and follow-up rates for each individual procedure are highlighted in Fig. 1.

The cohort comprised 71 women (64.0%) and 40 men (36.0%). Median age was 67.2 (range 65.0–76.7.0.7) years, mean weight at the time of the operation was 125.8 ± 22.3 kg, resulting in a mean BMI of 45.2 ± 7.4 kg/$\:{\mathrm{m}}^{2}$, and a mean excess weight of 56.1 ± 20.1 kg (Table 1).

**Table 1:** Baseline characteristics

	All patients (n) = 111
Age (years)	67.2 (*R* 65.0–76.7.0.7)
Sex, f.	71 (64.0%)
Sex, m.	40 (36.0%)
Height (cm)	167 ± 10.0
**Baseline weight characteristics**:	
Weight (kg)	125.8 ± 22.3
BMI (kg/)	45.2 ± 7.4
Ideal weight (kg)*	69.7 ± 7.1
Excess weight (kg)	56.1 ± 20.1
**Operations**:	
SG (n, %)	12 (10.8)
RYGB (n, %)	40 (36.0)
OAGB (n, %)	41 (36.9)
SADI-S (n, %)	18 (16.2)

Body-Mass-Index (*BMI*), Sleeve Gastrectomy (*SG*), Roux-en-Y-Gastric Bypass (*RYGB*), One Anastomosis Gastric Bypass (*OAGB*), Single Anastomosis Duodeno-ileal Bypass with Sleeve (SADI-S)

*Based on BMI 25.0kg/m^2^

The procedures performed were SG in 12 (10.8%), RYGB in 40 (36.0%), OAGB in 41 (36.9%), and SADI-S in 18 (16.2%) patients (see Table 1). Follow-up rates were 80.2% at 1 year, 72.7% at 2 years, and 77.3% at 5 years.

### Weight Loss Outcomes Overall

Baseline weight and BMI and weight-related outcomes are presented in Table 2.

**Table 2:** Weight loss related outcomes

	Baseline (*n* = 111)	1-year FU (*n* = 84)	2-year FU (*n* = 70)	5-year FU (*n* = 48)
Weight (kg)	125.8 ± 22.3 127.0 [27.9]	91.0 ± 17.4 90.5 [23.6]	84.0 ± 17.6 80.0 [23.0]	77.9 ± 15.4 79.0 [20.5]
BMI (kg/$\:{\boldsymbol{m}}^{2}$)	45.2 ± 7.4 44.6 [8.5]	32.8 ± 6.1 32.7 [7.8]	30.4 ± 6.1 30.1 [8.2]	27.5 ± 6.2 27.6 [5.9]
BMI change		−12.5 ± 6.3 −11.6 [9.1]	−14.5 ± 6.4 −14.3 [8.6]	−17.9 ± 7.6 16.5 [6.7]
Total weight loss, TWL; (%)		26.8 ± 12.3 31.6 [23.6]	31.7 ± 12.2 40.0 [24.5]	37.5 ± 14.7 45.5 [22.9]
Excess weight loss, EWL; (%)		67.7 ± 44.6 63.6 [31.9]	76.4 ± 27.5 75.7 [29.0]	89.7 ± 31.3 87.1 [32.0]

Data is presented using mean ± standard deviation and median [interquartile range]

Follow Up (*FU*), Body-Mass-Index (*BMI*), Total weight loss (*TWL*), excess weight loss (*EWL*)

Mean weight decreased from 125.8 ± 22.3 kg at baseline to 91.0 ± 17.4 kg at 1 year, 84.0 ± 17.6 kg at 2 years, and 77.9 ± 15.4 kg at 5 years. This corresponds to a reduction in BMI from 45.2 ± 7.4 kg/m^2^ to 32.8 ± 6.1, 30.4 ± 6.1, and 27.5 ± 6.2 kg/m^2^, respectively (Table 2).

### Weight Loss Outcomes by Procedure

BMI, TWL, and EWL stratified by procedure are shown in Table 3.

**Table 3:** Body-Mass-Index (BMI) and total weight loss (TWL) stratified by procedure

		Baseline (*n* = 111)		1-year FU (*n* = 84)	2-year FU (*n* = 70)	5-year FU (*n* = 48)
**RYGB**	BMI		42.6 ± 7.6 43.3 [7.7]	30.6 ± 5.2 30.7 [7.3]	28.9 ± 3.9 29.1 [5.5]	26.8 ± 3.8 27.4 [3.8]
	**TWL** **EWL**			26.0 ± 14.1 31.0 [18.0] 79.4 ± 62.7 71.72 [34.2]	29.7 ± 12.3 36.0 [20.6] 80.4 ± 23.6 81.0 [27.5]	35.9 ± 13.2 45.0 [20.5] 95.6 ± 28.2 87.7 [23.4]
**OAGB**	**BMI**		44.2 ± 5.2 43.9 [6.8]	31.1 ± 4.9 31.2 [4.8]	27.5 ± 4.4 26.8 [4.6]	28.1 ± 5.2 27.8 [6.2]
	**TWL** **EWL**			29.1 ± 9.5 33.0 [18.3] 69.3 ± 24.2 64.6 [22.7]	35.5 ± 8.1 42.4 [20.3] 88.6 ± 22.9 88.0 [23.6]	36.2 ± 10.2 44.0 [23.5] 85.9 ± 26.2 87.0 [35.1]
**SADI-S**	**BMI**		52.51 ± 5.0 52.2 [6.5]	36.1 ± 5.8 36.8 [4.5]	33.2 ± 6.6 34.0 [8.3]	29.3 ± 6.4 29.3 [9.1]
	**TWL** **EWL**			30.6 ± 12.1 43.4 [21.1] 58.8 ± 23.1 62.5 [19.1]	35.7 ± 13.1 52.2 [29.2] 69.5 ± 26.0 70.2 [29.5]	43.8 ± 17.0 43.8 [24.1] 82.2 ± 25.9 82.2 [52.4]
**SG**	**BMI**		46.4 ± 9.2 45.0 [10.4]	40.7 ± 5.8 41.4 [8.3]	40.0 ± 6.3 389 [7.6]	33.1 ± 5.0 32.4 [4.9]
	**TWL** **EWL**			15.1 ± 5.1 15.9 [10.6] 32.7 ± 9.4 37.0 [14.1]	14.4 ± 5.1 17.0 [11.1] 32.6 ± 11.8 34.3 [18.4]	27.7 ± 14.4 46.0 [18.0] 60.0 ± 29.6 70.8 [28.1]

Data is presented as mean ± standard deviation and median [interquartile range] in kg/m^2^ $\wedge$ %

Body-Mass-Index (*BMI*), Roux-en-Y-Gastric Bypass (*RYGB*), One Anastomosis Gastric Bypass (*OAGB*), Single Anastomosis Duodeno-ileal Bypass with Sleeve (*SADI-S*), follow-up (*FU*)

**Fig. 2:**
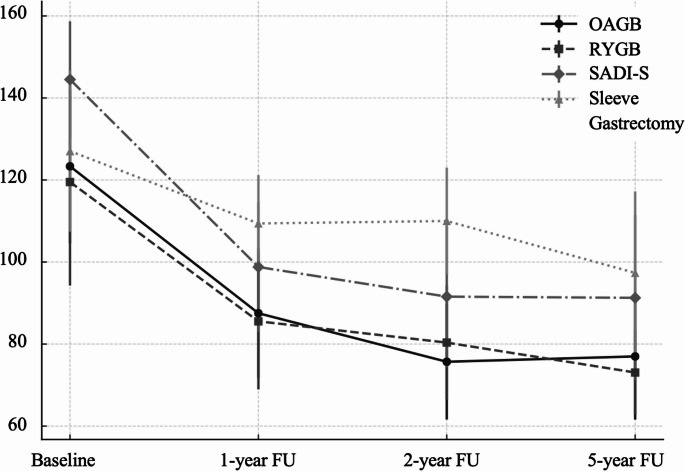
Mean body weight stratified by procedure over time. Data is presented in kg. Patients included at FU timepoints: 1-year (n=84), 2-year (n=70), 5-year (n=48). Abbreviations: Follow Up (FU), Omega Loop Bypass (OAGB), Roux-en-Y Bypass (RYGB), Single Anastomosis Duodenoileal Bypass with Sleeve (SADI-S), Sleeve Gatrectomy (SG)

At baseline, mean BMI was highest for SADI-S with 52.5 ± 5.0 kg/$\:{\mathrm{m}}^{2}\:\:$and lowest for RYGB with 42.6 ± 7.62 kg/$\:{\mathrm{m}}^{2}$. At five years, mean BMI was lowest for SADI-S with 26.78 ± 3.8 kg/$\:{\mathrm{m}}^{2}$ and highest for SG with 33.1 ± 5.0 kg/$\:{\mathrm{m}}^{2}$. Mean TWL at 5 years was highest for SADI-S 43.8 ± 17.0% and lowest for SG with 27.7 ± 14.4%. Overall, all procedures resulted in an increased TWL at each follow-up timepoint (Table 3). Figure 2 highlights the mean body weight over five years of follow-up, stratified by procedure.

### Obesity-Related Complications

**Fig. 3:**
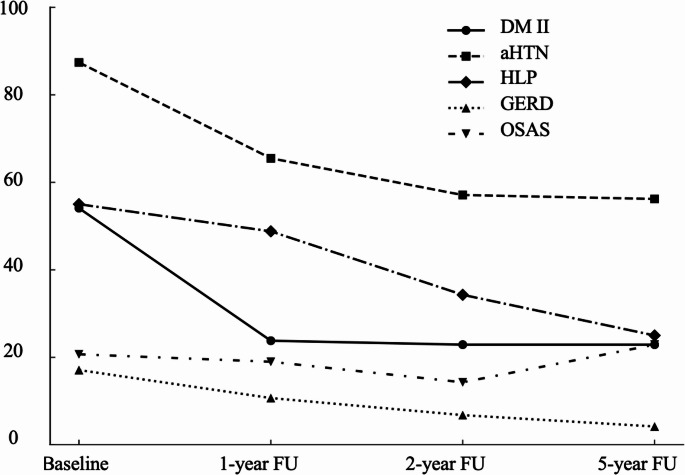
Associated medical conditions. Data is presented in absolute numbers and %. Patients included at FU timepoints: 1-year (n=84), 2-year (n=70), 5-year (n=48). Abbreviations: Follow Up (FU), Diabetes mellitus type II (DMII), arterial hypertension (aHTN), hyperlipidemia (HLP), obstructive sleep apnea syndrome (OSAS), gastro-esophageal reflux disease (GERD)

Baseline status on obesity-related complications is presented in Table 4 and reduced rates of obesity-associated medical conditions after one, two, and five years are presented in Table 4; Fig. 3. All surgical procedures led to an improvement in obesity-associated medical problems after one year, with prevalence decreasing further after 2 and 5 years for all conditions besides GERD (Table 4). GERD prevalence slightly increased from 20.7% at baseline to 22.9%. After five years, prevalence of obesity-related complications was at 57.7% for DMII, 35.7% for aHTN, 54.5% for HLP, and 75.4% for OSAS.

**Table 4:** Associated medical problems

	Baseline	1-year FU	2-year FU		5-year FU
	*n* = 111 (%)	*n* = 84 (%)	*n* = 70 (%)		*n* = 48 (%)
DMII	60 (54.1)	20 (23.8)	15 (22.9)	11 (22.9)	
aHTN	97 (87.4)	55 (65.5)	40 (57.1)	27 (56.2)	
HLP	61 (55.0)	41 (48.8)	24 (34.3)	12 (25.0)	
OSAS	19 (17.1)	9 (10.7)	6 (8.6)	2 (4.2)	
GERD	23 (20.7)	16 (19.0)	14 (20.0)	11 (22.9)	

Data is presented in absolute numbers and %

Follow Up (*FU*), Diabetes mellitus type II (*DMII*), arterial hypertension (*aHTN*), hyperlipidemia (*HLP*), Obstructive sleep apnea syndrome (*OSAS*), Gastro-esophageal reflux disease (*GERD*)

### Complications and Revisional Procedures

Early complications (< 30 days) occurred in eight patients (7.2%), all of them Clavien Dindo 3b. Three patients presented with leaks (two with RYGB, one with SADI-S), two patients (both with SADI-S) suffered from gastrointestinal bleedings, two patients (RYGB, SG) developed abscesses in the surgical field and one patient had dysphagia (RYGB). All patients with early complications underwent reoperation, one patient with dysphagia was treated with an esophageal stent first. Six patients were treated within their initial stay and two were readmitted within the first 30 days. Four patients had to stay at the intensive care unit postoperatively for 2–8 days.

Midterm complications (> 30 days to 5 years) occurred in eight patients (7.2%), all of them were Clavien Dindo 3b, comprising four anastomotic ulcers (two patients each with OAGB and RYGB), one incisional hernia (one patient with OAGB), one ileus due to a Peterson space’s herniation (in a patient with RYGB), and one dysphagia (in a patient with SADI-S). Patients with anastomotic ulcers were diagnosed by endoscopy and treated conservatively first before having a reoperation. The patients with Peterson space hernia and incisional hernia were diagnosed by computed tomography and immediately admitted to reoperation. The patient with dysphagia after SADI-S was diagnosed clinically by gastroscopy and was treated by stenting. Overall, reoperation due to complications was required in ten patients (9.0%). Restoration to normal anatomy was performed in two patients (1.8%), one was due to malnutrition after OAGB and the other one due to vitamin and calcium deficiency after RYGB.

Slightly higher complication rates were observed for SADI-S and RYGB compared to SG and OAGB. Nevertheless, due to the small sample size in the subgroups and low event numbers, no general conclusion can be drawn. A total of twelve deaths (10.8%) occurred during the follow-up period of this study. None occurred within one year after MBS and none was MBS-related. Reasons for death were myocardial infarction (*n* = 1), heart failure (*n* = 2), respiratory failure (*n* = 2), sepsis (*n* = 1), cerebral bleeding (*n* = 2), carcinomas (*n* = 2), and unknown (*n* = 2).

## Discussion

This study represents one of the largest European single-center cohorts evaluating MBS outcomes specifically in patients aged ≥ 65years, with meaningful mid-term follow-up up to five years. Our findings demonstrate that in carefully selected elderly patients, MBS results in clinically meaningful and durable weight loss, accompanied by substantial improvement or remission of obesity-related medical conditions. Although complication rates are higher than those reported in younger populations in the literature, the overall benefit-risk profile remains favorable.

### Weight-Loss Outcomes

In elderly patients, effective early weight loss is of particular importance as it contributes to the reduction of obesity-related complications, musculoskeletal pain, and functional impairment, and thereby improves mobility and quality of life.

In this study, all procedures resulted in substantial weight loss. At five years, the majority of patients achieved a postoperative BMI < 30 kg/m^2^, with the exception of those who had SG. TWL and EWL were broadly comparable to outcomes in younger cohorts, confirming that advanced age alone does not preclude effective metabolic results [13]. Range and IQR of EWL and TWL in the present study showed clearly that in all surgical groups, the majority of patients do not present with potentially concerning results such as very low BMI with possible undernutrition or persistent severe obesity.

To our knowledge, there is currently no published literature considering SADI-S in patients aged 65 years or above. SADI-S showed excellent weight loss results in this study, even comparable to weight loss results in younger patients [14]. These findings suggest that advanced age alone should not automatically preclude the use of SADI-S in carefully selected elderly patients. Further, SADI-S is known to have the best remission rates of T2DM [13], so existent T2DM might strengthen the recommendation of this procedure for elderly individuals.

Consistent with the literature available, SG demonstrated relatively lowlong-term weight loss durability [15] compared to the bypass procedures (OAGB and RYGB) [16, 17]. SG is often favored due to its perceived lower surgical complications compared to RYGB and OAGB in patients with obesity grade 3 [18], obesity grade > 4 [19], and in elderly patients [20]. However, this advantage must be weighed against its reduced long-term weight loss and the higher risk of mid- and long-term weight regain. The clinical relevance of recurrent weight gain several years after MBS might not be as important as a lower complication rate in elderly patients when choosing the procedure. Nevertheless, long-term durability remains a key consideration.

While short-term BMI reduction after SG is comparable to RYGB and OAGB [21, 22], bypass procedures demonstrate superior long-term weight loss. Randomized data has shown no significant difference between SG and RYGB at five years, but a clear advantage for RYGB at ten years, and long-term conversion rates after SG of up to 50% due to weight regain or reflux have been reported [15, 17].

Slightly better weight-loss outcomes have been reported for OAGB compared to RYGB in some studies [13, 20], a trend also observed in our cohort. However, randomized data from the Yomega trial, the only RCT comparing OAGB und RYGB to our knowledge, showed no significant difference regarding weight loss between OAGB and RYGB after five years [23]. As weight loss outcomes are influenced by biliopancreatic limb length, which varies considerably between studies, OAGB and RYGB should be regarded as largely equivalent in terms of weight loss efficacy, including in elderly patients. Consequently, procedure selection should be individualized.

### Remission of Obesity-Associated Medical Conditions

MBS is a highly effective intervention not only for sustained weight loss but also for the improvement and remission of obesity-associated medical conditions. Consistent with the existing literature, remission rates for T2DM, aHTN, and HLP are substantial. A systematic review by Balamurugan et al. among patients of all ages reported remission rates exceeding 73%, 60%, and 82% for T2DM, aHTN, and HLP after RYGB, and 76%, 64%, and 71% after OAGB, respectively [13].

Given that HLP and aHTN are generally more prevalent and often have persisted over a longer time in elderly patients, slightly lower remission rates compared with cohorts including patients of all ages are expected and have been reported previously [24, 25].

Interestingly, remission of OSAS was higher in this study than reported in comparable series [26]. This finding suggests that preexisting OSAS in elderly patients should be regarded as a strong argument in favor of MBS, even later in life.

In contrast, GERD was the only obesity-associated complication that increased slightly after MBS in the present study. This observation is likely multifactorial and consistent with prior evidence, showing that MBS – particularly SG – may induce de-novo GERD or worsen pre-existing reflux symptoms, often necessitating PPI therapy. The SLEEVEPASS RCT [27] demonstrated a significantly higher rate of GERD after SG compared with RYGB. Similarly, worsening of pre-existing or de-novo manifestation of GERD is also reported after SADI-S [14], likely attributable to the intact pylorus, resulting in a high-pressure system in the gastric sleeve. Further, OAGB may increase the risk for biliary reflux, which is often asymptomatic but can lead to chronic inflammation at the gastroesophageal junction [28].

To choose the best procedure for every elderly individual, GERD must be assessed preoperatively via clinical examination and gastroscopy in all (symptomatic and asymptomatic) patients [29, 30]. If patients present with GERD at the MUV, RYGB is usually the preferred procedure. Routine post-surgery gastroscopy after MBS is advised according to the IFSO consensus guidelines of 2025 if patients develop altered symptoms such as dysphagia, odynophagia, weight loss, anaemia, or gastrointestinal bleeding of refractory GERD [31].

To sum up, the remission rate of obesity-associated medical problems after MBS in the elderly was high with a slight increase of GERD at the end of the follow-up. Thus, patients above 65 years of age may benefit from MBS in this regard.

Interestingly, attenuation of weight loss and resolution of obesity-related complications over a period of five years was not observed in this series of elderly patients. Reasons for this phenomenon can only be hypothetical here, but may be explained by patient selection as, of course, only a minority of (very motivated) elderly patients would undergo MBS. Further, the occurrence of receiving medical support in this cohort of elderly patients is high as a majority has other diseases as well, with the need for checks and treatment at regular intervals, which probably leads to better overall compliance.

Finally, conditions that are also important but were not assessed in this study are bone health, muscle strength, and the potential risk of sarcopenia. These conditions could be future research topics when studying elderly patients after MBS.

### Complications

Complication rates in our study of elderly patients are slightly higher compared to studies with adults of all ages in the literature and compared to the outcome of adults in our center [15]. Importantly, this outcome appears to be driven less by chronological age alone and more by the higher burden and severity of obesity-related medical problems in this population. Additionally, many elderly patients receive chronic anticoagulation or antiplatelet therapy, which are changed to low-molecular weight heparin when undergoing surgery. This increases the risk of perioperative bleeding in patients of all ages as known from the literature [32]. Our findings are consistent with a larger Scandinavian registry study by Gerber at al., which analyzed 47,660 patients of all ages undergoing primary gastric bypass and demonstrated an elevated overall complication rate of 10.0–11.0% in elderly patients compared to 8–9% in adults of all ages. Anastomotic leakage rates were also higher in the elderly subgroup [33].

Mid-term complications in this study occurred in eight patients, with marginal (anastomotic) ulcers accounting for half of these cases, requiring surgical revision. All patients received routine PPI therapy for at least 3 months (extended to 12 months in smokers). This observed rate of marginal ulcers is comparable to previously reported incidences of up to 16% in the literature [31]. Smoking remains one of the most important risk factors for marginal ulcer formation [31]. While some countries consider ongoing smoking a contraindication to MBS, in Austria, surgery is not withheld from patients who are unable to quit smoking, accepting a higher risk of marginal ulceration in favor of providing effective obesity treatment. Other late complications, including incisional hernia, ileus, and dysphagia, were reported one case each and were comparable to rates reported in adult populations of all ages [27].

A total of twelve deaths occurred during follow-up; however, none were related to the bariatric procedure itself. As expected, overall mortality during long-term follow-up was higher when compared to younger cohorts, reflecting reduced life expectancy and a higher burden of associated medical conditions in elderly patients. Nevertheless, existing evidence suggests that MBS is associated with an overall survival benefit even in patients older than 65 years, supporting its role as a life-prolonging intervention in carefully selected patients [34].

### Limitations

The retrospective design is a limitation of this study. The absence of follow-up gastroscopies leads to an incomplete picture regarding the effects of GERD in this cohort of elderly patients. Still, a detailed clinical assessment of GERD was performed.

Nevertheless, the data of this study can still be compared to the existing literature. Additionally, the relatively high follow-up rate leads to a high reliability of this study. Due to the low number of patients (and therefore only descriptive statistics), a comparison between the different procedures regarding their efficacy cannot be drawn. Further, survival curves or survival rates by year were not possible due to limited data.

Unfortunately, perioperative risk profiles (including ASA score, frailty assessment, Charlson Comorbidity Index, smoking status, anticoagulation, and functional status) were not available for the majority of patients as this information is not available especially for the operations performed several years ago. Also, this study provides no clear criteria for the selection of patients if they were not healthy enough to undergo the anesthesiologic risk of an MBS as this was decided on individual bases.

Further, occurrence of attrition bias, i.e., patients with poorer outcomes may be less likely to attend follow-up, cannot be ruled out completely.

Another important limitation of this study is that no assessment regarding bone health, muscle strength, sarcopenia or quality of life score was done as the majority of the elderly patients were not willing to undergo any further examinations. Also, diagnosis of GERD was only done clinically and not by follow-up endoscopy.

## Conclusion

MBS is safe and effective in selected elderly patients, yielding meaningful weight loss and remission of obesity-related medical conditions. Although complication rates tend to be relatively high, the overall benefit-risk ratio supports using MBS for appropriately selected individuals ≥ 65 years without severe medical conditions that would increase perioperative risk.

## Data Availability

Data was generated using for medical purposes routinely collected patient data. Data was anonymized and transferred to an Excel spreadsheet.

## References

[1] Kloock S, Ziegler CG, Dischinger U. Obesity and its comorbidities, current treatment options and future perspectives: Challenging bariatric surgery? Pharmacol Ther. 2023;251:108549. 10.1016/j.pharmthera.2023.108549. 37879540 10.1016/j.pharmthera.2023.108549

[2] Arterburn DE, Telem DA, Kushner RF, Courcoulas AP. Benefits and risks of bariatric surgery in adults: A review. JAMA. 2020;324(9):879–87. 10.1001/jama.2020.12567. 32870301 10.1001/jama.2020.12567

[3] Angrisani L, Santonicola A, Iovino P, Palma R, Kow L, Prager G, et al. IFSO worldwide survey 2020–2021: Current trends for bariatric and metabolic procedures. Obes Surg. 2024;34(4):1075–85. 10.1007/s11695-024-07118-3. 38438667 10.1007/s11695-024-07118-3 PMC11026210

[4] Caroline M, Apovian MD, Obesity. Definition, Comorbidities, Causes, and Burden [Internet]. Vol. 22. 2016 Jun 2 [cited 2025 Jul 12];Impact of Obesity Interventions on Managed Care22. Available from: https://www.ajmc.com/view/obesity-definition-comorbidities-causes-burden 27356115

[5] Karlamangla A, Tinetti M, Guralnik J, Studenski S, Wetle T, Reuben D. Comorbidity in older adults: Nosology of impairment, diseases, and conditions. J Gerontol A Biol Sci Med Sci. 2007;62(3):296–300. 10.1093/gerona/62.3.296. 17389727 10.1093/gerona/62.3.296

[6] Goldberg I, Yang J, Nie L, Bates AT, Docimo S, Pryor AD, et al. Safety of bariatric surgery in patients older than 65 years. Surg Obes Relat Dis. 2019;15(8):1380–7. 10.1016/j.soard.2019.05.016. 31248793 10.1016/j.soard.2019.05.016

[7] Yim THJYZ, Tan KY. Functional outcomes after abdominal surgery in older adults - How concerned are we about this? Eur J Surg Oncol. 2024;50(6):108347. 10.1016/j.ejso.2024.108347. 38657374 10.1016/j.ejso.2024.108347

[8] Impact of Age on Perioperative Complications and Length of Stay in Patients Undergoing. Noncardiac Surgery | Annals of Internal Medicine [Internet]. [cited 2025 Jul 12]. Available from: https://www.acpjournals.org/doi/full/10.7326/0003-4819-134-8-200104170-00008 10.7326/0003-4819-134-8-200104170-00008 11304103

[9] Felsenreich DM, Bichler C, Langer FB, Gachabayov M, Prager G. Sleeve Gastrectomy: Surgical Technique, Outcomes, and Complications. Surg Technol Int. 2020;36:63–9. PubMed PMID: 32359172. 32359172

[10] Felsenreich DM, Langer FB, Bichler C, Eichelter J, Jedamzik J, Gachabayov M, et al. Surgical technique of diverted one-anastomosis gastric bypass. Surg Technol Int. 2021;39:107–12. 10.52198/21.STI.39.GS1485. 34699605

[11] Felsenreich DM, Bichler C, Langer FB, Gachabayov M, Eichelter J, Gensthaler L, et al. Surgical Technique for One-Anastomosis Gastric Bypass. Surg Technol Int. 2020;37:57–61. PubMed PMID: 33180956. 33180956

[12] Eichelter J, Felsenreich DM, Bichler C, Gensthaler L, Gachabayov M, Richwien P, et al. Surgical technique of single anastomosis duodeno-ileal bypass with sleeve gastrectomy (SADI-S). Surg Technol Int. 2022;41:111–7. 10.52198/22.STI.41.GS1571. 35623034 10.52198/22.STI.41.GS1571

[13] Balamurugan G, Leo SJ, Sivagnanam ST, Balaji Prasad S, Ravindra C, Rengan V, et al. Comparison of efficacy and safety between Roux-en-Y gastric bypass (RYGB) vs one anastomosis gastric bypass (OAGB) vs single anastomosis duodeno-ileal bypass with sleeve gastrectomy (SADI-S): a systematic review of bariatric and metabolic surgery. Obes Surg. 2023;33(7):2194–209. 10.1007/s11695-023-06602-6. 37140720 10.1007/s11695-023-06602-6

[14] Ponce de Leon-Ballesteros G, Romero-Velez G, Higa K, Himpens J, O’ Kane M, Torres A, et al. Single anastomosis duodeno-ileostomy with sleeve gastrectomy/single anastomosis duodenal switch (SADI-S/SADS) IFSO position statement—update 2023. Obes Surg. 2024;34(10):3639–85. 10.1007/s11695-024-07490-0. 39264553 10.1007/s11695-024-07490-0

[15] Felsenreich DM, Artemiou E, Steinlechner K, Vock N, Jedamzik J, Eichelter J, et al. Fifteen years after sleeve gastrectomy: weight loss, remission of associated medical problems, quality of life, and conversions to Roux-en-Y gastric bypass—long-term follow-up in a multicenter study. Obes Surg. 2021;31(8):3453–61. 10.1007/s11695-021-05475-x. 34021882 10.1007/s11695-021-05475-x PMC8270807

[16] O’Brien PE, Hindle A, Brennan L, Skinner S, Burton P, Smith A, et al. Long-term outcomes after bariatric surgery: a systematic review and meta-analysis of weight loss at 10 or more years for all bariatric procedures and a single-centre review of 20-year outcomes after adjustable gastric banding. Obes Surg. 2019;29(1):3–14. 10.1007/s11695-018-3525-0. 30293134 10.1007/s11695-018-3525-0 PMC6320354

[17] Kraljević M, Süsstrunk J, Wölnerhanssen BK, Peters T, Bueter M, Gero D, et al. Long-term outcomes of laparoscopic Roux-en-Y gastric bypass vs laparoscopic sleeve gastrectomy for obesity: The SM-BOSS randomized clinical trial. JAMA Surg. 2025;160(4):369–77. 10.1001/jamasurg.2024.7052. 39969869 10.1001/jamasurg.2024.7052 PMC11840683

[18] Alaidaroos O, Al Jaber AA, Al Jaber AA, Alshehri AH, Alkehaimi MB, Alsannat OA. Long-term outcomes of sleeve gastrectomy versus gastric bypass. Cureus. 2024;16(11):e72961. 10.7759/cureus.72961. 39498430 10.7759/cureus.72961 PMC11533043

[19] Soong TC, Lee MH, Lee WJ, Almalki OM, Chen JC, Wu CC, et al. Long-term efficacy of bariatric surgery for the treatment of super-obesity: comparison of SG, RYGB, and OAGB. Obes Surg. 2021;31(8):3391–9. 10.1007/s11695-021-05464-0. 33993423 10.1007/s11695-021-05464-0

[20] Dowgiałło-Gornowicz N, Lech P, Major P. Bariatric and metabolic surgery in patients older than 65 years – a multicenter study. Obes Surg. 2023;33(10):3106–11. 10.1007/s11695-023-06750-9. 37566339 10.1007/s11695-023-06750-9 PMC10514098

[21] Cui B, Zhu L, Zhu S. Effects of Roux-en-Y gastric bypass versus sleeve gastrectomy on body composition for patients with a BMI > 35 kg/m2 at 1 year after surgery. Obes Surg. 2022;32(5):1658–66. 10.1007/s11695-022-06006-y. 35294693 10.1007/s11695-022-06006-y

[22] Delko T, Kraljević M, Lazaridis II, Köstler T, Jomard A, Taheri A, et al. Laparoscopic Roux-Y-gastric bypass versus laparoscopic one-anastomosis gastric bypass for obesity: clinical & metabolic results of a prospective randomized controlled trial. Surg Endosc. 2024;38(7):3875–86. 10.1007/s00464-024-10907-7. 38831218 10.1007/s00464-024-10907-7

[23] Robert M, Poghosyan T, Maucort-Boulch D, Filippello A, Caiazzo R, Sterkers A, et al. Efficacy and safety of one anastomosis gastric bypass versus Roux-en-Y gastric bypass at 5 years (YOMEGA): a prospective, open-label, non-inferiority, randomised extension study. Lancet Diabetes Endocrinol. 2024;12(4):267–76. 10.1016/S2213-8587(24)00035-4. 38452784 10.1016/S2213-8587(24)00035-4

[24] Rosada A, Kassner U, Weidemann F, König M, Buchmann N, Steinhagen-Thiessen E, et al. Hyperlipidemias in elderly patients: results from the Berlin Aging Study II (BASEII), a cross-sectional study. Lipids Health Dis. 2020;19(1):92. 10.1186/s12944-020-01277-9 . PubMed PMID: 32410691; PubMed Central PMCID: PMC7227351. 32410691 10.1186/s12944-020-01277-9 PMC7227351

[25] Fagard RH. Epidemiology of hypertension in the elderly. Am J Geriatr Cardiol. 2002;11(1):23–8. 10.1111/j.1076-7460.2002.00856.x. 11773712 10.1111/j.1076-7460.2002.00856.x

[26] Al Oweidat K, Toubasi AA, Tawileh RBA, Tawileh HBA, Hasuneh MM. Bariatric surgery and obstructive sleep apnea: a systematic review and meta-analysis. Sleep Breath. 2023;27(6):2283–94. 10.1007/s11325-023-02840-1. 37145243 10.1007/s11325-023-02840-1

[27] Salminen P, Grönroos S, Helmiö M, Hurme S, Juuti A, Juusela R, et al. Effect of laparoscopic sleeve gastrectomy vs Roux-en-Y gastric bypass on weight loss, comorbidities, and reflux at 10 years in adult patients with obesity. JAMA Surg. 2022;157(8):656–66. 10.1001/jamasurg.2022.2229. 35731535 10.1001/jamasurg.2022.2229 PMC9218929

[28] Szymański M, Marek I, Wilczyński M, Janczy A, Bigda J, Kaska Ł, et al. Evaluation of esophageal pathology in a group of patients 2 years after one-anastomosis gastric bypass (OAGB) — cohort study. Obes Res Clin Pract. 2022;16(1):82–6. 10.1016/j.orcp.2021.12.001. 34922847 10.1016/j.orcp.2021.12.001

[29] Abdallah H, El Skalli M, Mcheimeche H, Casagranda B, de Manzini N, Palmisano S. Indications for upper gastrointestinal endoscopy before bariatric surgery: a multicenter study. Surg Endosc. 2023;37(2):1342–8. 10.1007/s00464-022-09656-2 . PubMed PMID: 36203110; PubMed Central PMCID: PMC9944709. 36203110 10.1007/s00464-022-09656-2 PMC9944709

[30] Brown WA, Johari Halim Shah Y, Balalis G, Bashir A, Ramos A, Kow L, et al. IFSO position statement on the role of esophago-gastro-duodenal endoscopy prior to and after bariatric and metabolic surgery procedures. Obes Surg. 2020;30(8):3135–53. 10.1007/s11695-020-04720-z. 32472360 10.1007/s11695-020-04720-z

[31] Vosburg RW, Nimeri A, Azagury D, Grover B, Noria S, Papasavas P, et al. ASMBS literature review on the treatment of marginal ulcers after metabolic and bariatric surgery. Surg Obes Relat Dis. 2025;21(1):1–8. 10.1016/j.soard.2024.10.003. 39516065 10.1016/j.soard.2024.10.003

[32] Markedly increased risk of postoperative bleeding. complications during perioperative bridging anticoagulation in general and visceral surgery - PMC [Internet]. [cited 2026 Feb 10]. Available from: https://pmc-ncbi-nlm-nih-gov.ez.srv.meduniwien.ac.at/articles/PMC7682086/10.1186/s13741-020-00170-4 PMC7682086 33292504

[33] Gerber P, Anderin C, Szabo E, Näslund I, Thorell A. Impact of age on risk of complications after gastric bypass: a cohort study from the Scandinavian Obesity Surgery Registry (SOReg). Surg Obes Relat Dis. 2018;14(4):437–42. 10.1016/j.soard.2017.12.024. 29428689 10.1016/j.soard.2017.12.024

[34] Belluzzi A, De Luca M, Monami M, Petry TBZ, Shikora SA, Cohen RV. Primary metabolic and bariatric surgery in persons aged over 65 years. GRADE-based International Federation for the Surgery of Obesity and Metabolic Disorders (IFSO) position statement. Obes Surg. 2025;35(11):4766–96. 10.1007/s11695-025-08113-y. 41042300 10.1007/s11695-025-08113-y

